# Data on clinical and economic burden associated with pulmonary arterial hypertension related hospitalizations in the United States

**DOI:** 10.1016/j.dib.2020.106303

**Published:** 2020-09-11

**Authors:** Abhishek Chaturvedi, Manreet Kanwar, Parul Chandrika, Thenappan Thenappan, Amresh Raina, Raymond L Benza

**Affiliations:** a Pauley Heart Center, Virginia Commonwealth University, Richmond, Virginia, United States; b Cardiovascular Institute, Allegheny General Hospital, Pittsburgh, PA, United States; c Department of Internal Medicine, Vidant Medical Center, Brody School of Medicine, Greenville, NC, United States; d Department of Cardiovascular Medicine, University of Minnesota, Minneapolis, MN, United States; e Division of Cardiovascular Medicine, The Ohio State University, Columbus, OH, United States

**Keywords:** Pulmonary arterial hypertension, National inpatient sample, Inpatient mortality, Multivariate analysis

## Abstract

A comprehensive description of the contemporary trends in pulmonary arterial hypertension (PAH) related hospitalizations, associated inpatient outcomes and predictors of worse outcomes were reported in our paper recently published in the International Journal of Cardiology [1]. Our observational analysis utilized ten year of national inpatient sample from January 1st 2007 through December 31st 2016. This Data in Brief companion paper aims to report the specific statistical highlights of the entire ten-year PAH cohort including demographics, hospital characteristics, regional variation, prevalence of comorbidities, and multivariable regression analysis used to examine the factors associated with increased inpatient mortality and prolonged length of stay. Additionally, we report trends in the cost (the actual amount of money reimbursed to the hospitals) of PAH related hospitalizations over the past ten years.

## Specifications Table

**Table:** 

Subject	Cardiology and Cardiovascular Medicine
Specific subject area	Pulmonary Hypertension
Type of data	Table Figure
How data were acquired	Data obtained from the official website of Healthcare Cost & Utilization Project and was the analysed by the authors using Stata 15.
Data format	Secondary data Analysed Descriptive
Parameters for data collection	Sample: National inpatient sample was used to identify patients with primary discharge diagnosis of pulmonary arterial hypertension using ICD-9 code 416.0 from 01/01/2007 through 09/30/2015 and ICD-10 code I27.0 from 10/01/2015 through 12/31/2016[2]. Records suggestive of secondary causes of pulmonary hypertension (WHO category II-V) were excluded. Parameters: Demographics, comorbidities, admission characteristics (elective vs. non-elective, primary payer, disposition status), and hospital characteristics (US region, government vs. private, teaching vs. non-teaching, hospital bed-size), income status, primary payer, discharge disposition, charges, costs, length of stay, all-cause mortality.
Description of data collection	National inpatient sample is the largest, publicly available, all-payer administrative claims database of inpatient hospitalizations in the US under Healthcare Cost & Utilization Project. It represents a random, 20% stratified sample of all inpatient hospitalizations from approximately 1000 non-federal hospitals in 46 states (representing > 97% of the total US population) and includes approximately 7 to 8 million hospitalizations per year [2].
Data source location	Agency for Healthcare Research and Quality, Rockville, MD, United States
Data accessibility	Analysed data are hosted with the article. Data of the NIS 2007–2016 are available to all researchers following a standard application process and signing of a data use agreement. National Inpatient Sample, Healthcare Cost and Utilization Project, Agency for Healthcare Research and Quality, Rockville, MD (https://www.hcup-us.ahrq.gov/nisoverview.jsp). Instructions for accessing the raw data: 1. Mandatory training module and HCUP data use agreement: Available at https://www.hcup-us.ahrq.gov/DUA/dua_508/DUA508version.jsp 2. Register for an account with HCUP: https://www.distributor.hcup-us.ahrq.gov/SpecialPages/Register.aspx 3. Request and purchase National Inpatient Sample data for relevant years.
Related research article	Chaturvedi A., Kanwar M., Chandrika P., Thenappan T., Raina A., Benza RL. National Trends and Inpatient Outcomes of Pulmonary Arterial Hypertension Related Hospitalizations – Analysis of the National Inpatient Sample Database [1].

## Value of the Data

• The data presented here provide real world statistics on the demographics, racial distribution, regional variation, comorbidities, economic burden and in-hospital outcomes in Pulmonary arterial hypertension (PAH) patients. These data would help clinicians appropriately risk stratify the hospitalized PAH patients
• Our additional analysis on the hospital characteristics, regional variation and insurance status of PAH-related hospitalizations may be useful to the insurance companies and policymakers by making them aware of the disparities in the economic burden and outcomes in these patients at the institutional and regional levels.
• The analysis reported in this Data in brief paper may hopefully promote and advocate for the need for continued research and developments of treatment strategies aimed at decreasing the hospitalizations, mortality and financial burden in PAH patients.
• The methods described in this study can be useful for future studies on PAH using national inpatient sample.

## 1 Data Description

Data included analysis of PAH-related hospitalizations from January 1st 2007 through December 31st 2016 as reported in the related article [1].

**Fig. 1:**
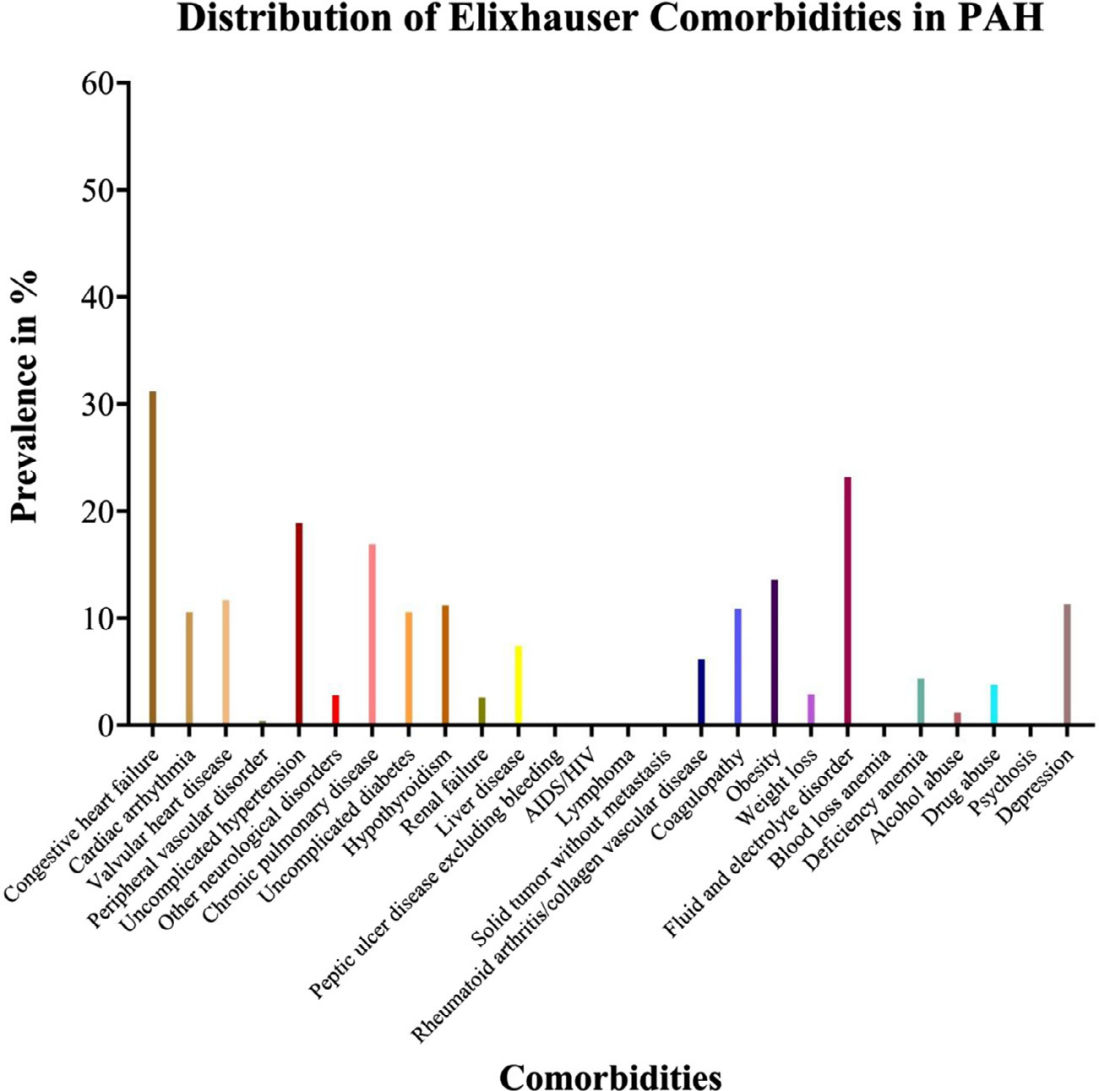
Elixhauser comorbidities in patients with pulmonary arterial hypertension.

**Fig. 2:**
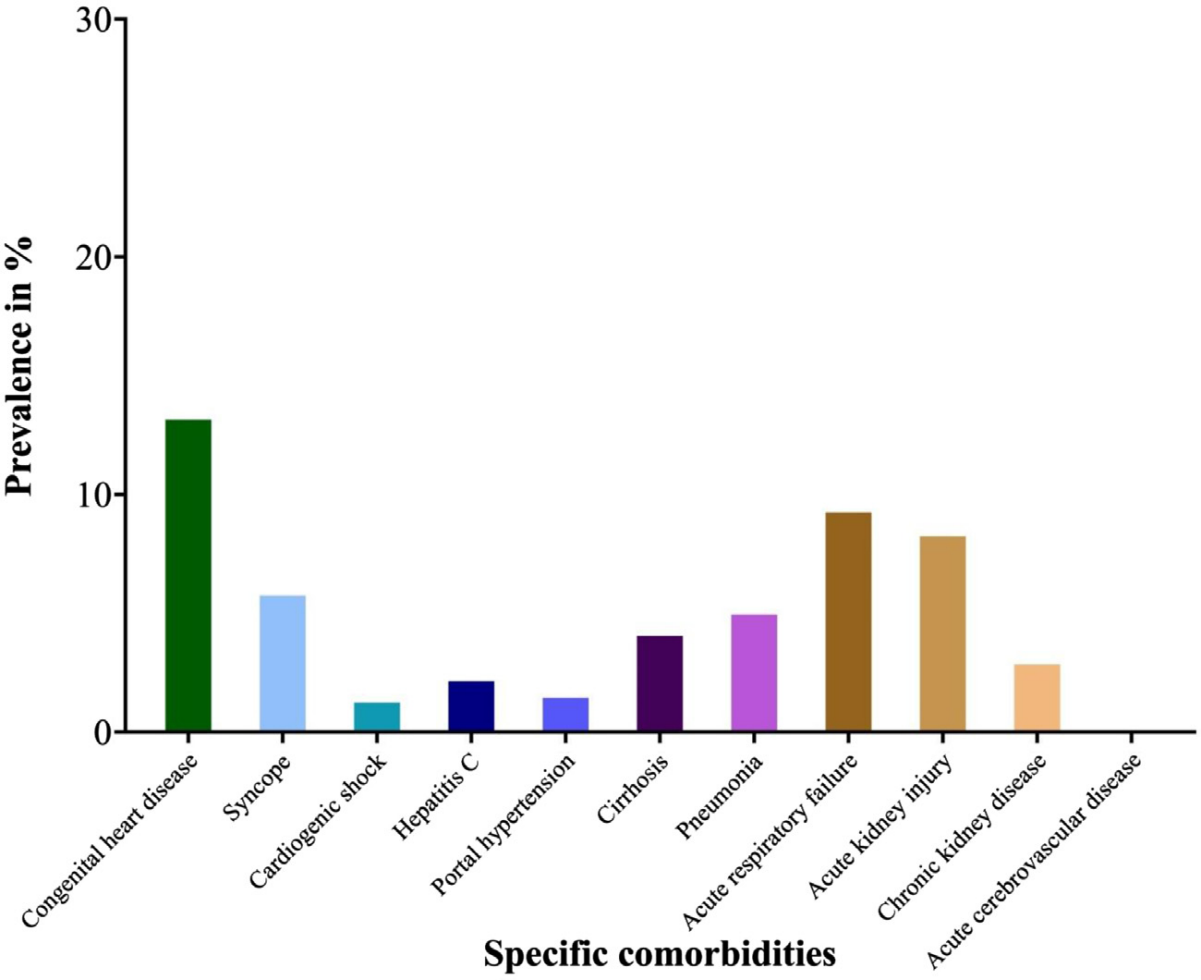
Other specific comorbidities in patients with pulmonary arterial hypertension.

Table 1 describes the overall demographics, comorbidities, hospital characteristics, regional variation, primary payer (insurance status) as well as inpatient outcomes including mortality, length of stay and charges (and costs) per PAH hospitalization. Mean age of the cohort was 38.7 years and 78.8% were female. Among these hospitalizations, 57.8% were Caucasians, 14.8% were African-Americans and 16.3% were Hispanic. Majority of patients were admitted to urban teaching (82.4%), private not-for-profit hospitals (78%) and large bed-size hospitals (76.1%). Patients were variably distributed among different US regions with highest proportion in South (31.4%) followed by West (29.7%), Northeast (22.8%) and Midwest (16.1%). Cohort had high comorbidity burden with mean Elixhauser comorbidity index 3.2. The prevalence of heart failure was highest (32%) followed by fluid and electrolyte disorders, uncomplicated hypertension, chronic pulmonary disease, obesity, congenital heart disease, valvular heart disease, depression, hypothyroidism, coagulopathy, arrhythmia, uncomplicated diabetes, acute respiratory failure and acute kidney injury (Figs. 1 and 2). Cohort had an overall IM of 6.03%, mean length of stay 7.6 days, mean inflation adjusted charges of $89,400 and mean inflation adjusted cost of $26,200.

**Table 1:** Hospitalization characteristics and inpatient outcomes in patients with pulmonary arterial hypertension.

Characteristic	Value
PAH discharges, *N*	6162
Age, years	38.7 ± 0.8
Female, %	78.8
Race*	
White, %	57.8
Black, %	14.8
Hispanic, %	16.3
Asian, %	3.5
Native American, %	2.2
**Location & teaching status**	
Rural, %	2.6
Urban nonteaching, %	15
Urban teaching, %	82.4
**Region**	
Northeast, %	22.8
Midwest, %	16.1
South, %	31.4
West, %	29.7
**Hospital control**	
Public, %	17.2
Private, not for profit, %	78
Private, for profit, %	4.8
**Hospital bed size**	
Small, %	6.4
Medium, %	17.5
Large, %	76.1
**Elixhauser comorbidities**	
Congestive heart failure, %	32
Cardiac arrhythmia, %	11.4
Valvular heart disease, %	12.5
Peripheral vascular disorder, %	1.2
Uncomplicated hypertension, %	19.7
Other neurological disorders, %	3.6
Chronic pulmonary disease, %	17.7
Uncomplicated diabetes, %	11.4
Hypothyroidism, %	12
Renal failure, %	3.4
Liver disease, %	8.2
Peptic ulcer disease excluding bleeding, %	0.4
AIDS/HIV, %	0.6
Lymphoma, %	0.4
Solid tumor without metastasis, %	0.5
Rheumatoid arthritis/collagen vascular disease, %	7
Coagulopathy, %	11.7
Obesity, %	14.4
Weight loss, %	3.7
Fluid and electrolyte disorder, %	24
Blood loss anemia, %	0.5
Deficiency anemia, %	5.2
Alcohol abuse, %	2
Drug abuse, %	4.6
Psychosis, %	0.3
Depression, %	12.1
Elixhauser comorbidity index	3.2 ± 0.1
**Other/specific comorbidities**	
Congenital heart disease, %	13.5
Syncope, %	6.1
Cardiogenic shock, %	1.6
Hepatitis C, %	2.5
Portal hypertension, %	1.8
Cirrhosis, %	4.4
Pneumonia, %	5.3
Acute respiratory failure, %	9.6
Acute kidney injury, %	8.6
Chronic kidney disease, %	3.2
Acute cerebrovascular disease, %	0.2
**Outcome measures**	
All-Cause inpatient mortality, %	6.03
Length of stay, days	7.6 ± 0.5
Total Charges, $ (x1,000)	84.1 ± 6.2
Inflation adjusted charges, $ (x1000)	89.4 ± 6.6
Total Cost, $ (x1,000)	24.6 ± 1.8
Inflation adjusted cost, $ (x1,000)	26.2 ± 1.9

Total number of PAH discharges are expressed as N. Continuous variables are expressed as mean ± SE and categorical variables are expressed as frequencies (%). PAH: Pulmonary arterial hypertension; SE: Standard error.

⁎ All the race categories shown in the table do not add up to 100% due to some patients being categorized as “other” in the NIS. Note: Pulmonary circulation disorder is an Elixhauser comorbidity that was not included in the analysis because PAH comes under this broad category of comorbidity.

Table 2 describes the multivariate analysis to identify predictors of inpatient mortality in patients with PAH related hospitalizations. The presence of right heart failure, cardiac arrhythmia, neurological disorders other than paralysis, fluid and electrolyte disorders, psychosis and increased length of stay were amongst factors associated with increased odds of inpatient mortality whereas younger age, Hispanic race and elective admission were associated with decreased odds of inpatient mortality.

**Table 2:** Multivariate regression analysis to determine predictors of inpatient mortality in pulmonary arterial hypertension patients.

Variable	Adjusted mortality OR (95% CI)	*p*
**Age, y**		
18–44	0.22 (0.10–0.46)	**<0.0001**
45–64	0.38 (0.15–0.95)	**0.038**
65–84	0.97 (0.38–2.48)	0.947
≥ 85	0.44 (0.04–4.47)	0.485
**Female gender**	0.74 (0.37–1.48)	0.391
**Owner**		
Government	Reference	NA
Private, Not for profit	0.69 (0.33–1.42)	0.307
Private, For profit	0.56 (0.14–2.28)	0.416
**Bed size**		
Small	Reference	NA
Medium	1.25 (0.40–3.94)	0.704
Large	0.97 (0.35–2.69)	0.947
**Race**		
White	Reference	NA
Black	0.93 (0.45–1.93)	0.84
Hispanic	0.31 (0.11–0.88)	**0.028**
Asian	1.64 (0.55–4.91)	0.377
Native American	0.71 (0.06–8.39)	0.784
**Region**		
Northeast	Reference	NA
Midwest	0.63 (0.20–1.97)	0.424
South	0.57 (0.26–1.23)	0.152
West	0.83 (0.36–1.94)	0.669
**Length of stay**	1.02 (1.01–1.03)	**0.005**
**Location**		
Rural location	Reference	NA
Urban, Non-teaching hospital	1.57 (0.13–18.39)	0.721
Urban, Teaching hospital	1.28 (0.14–11.98)	0.829
**Quartile classification of median household income for patient's ZIP Code**		
Quartile 1	Reference	NA
Quartile 2	0.96 (0.43–2.12)	0.909
Quartile 3	0.56 (0.26–1.20)	0.137
Quartile 4	0.48 (0.20–1.15)	0.099
**Admission type**		
Non-elective	Reference	NA
Elective	0.35 (0.14–0.90)	**0.029**
**Admission Day**		
Weekday	Reference	NA
Weekend	1.54 (0.68–3.48)	0.295
**Elixhauser comorbidities**		
Congestive heart failure	2.48 (1.35–4.58)	**0.004**
Cardiac arrhythmia	2.90 (1.49–5.66)	**0.002**
Peripheral vascular disorder	1.41 (0.16–12.67)	0.76
Other neurological disorders	3.33 (1.09–10.15)	**0.034**
Chronic pulmonary disease	0.73 (0.32–1.67)	0.452
Uncomplicated diabetes	0.71 (0.26–1.92)	0.501
Renal failure	1.11 (0.20–6.34)	0.904
Liver disease	0.66 (0.23–1.86)	0.428
Coagulopathy	0.91 (0.43–1.92)	0.804
Obesity	1.09 (0.43–2.75)	0.853
Weight loss	0.92 (0.17–5.06)	0.922
Fluid and electrolyte disorder	3.04 (1.56–5.93)	**0.001**
Alcohol abuse	2.76 (0.50–15.28)	0.246
Psychosis	14.81 (2.75–79.67)	**0.002**
Depression	0.42 (0.14–1.23)	0.113

*P* < 0.05 was considered statistically significant. Bolded text represents the subheadings within the table and bolded numbers represent *p* < 0.05. OR- Odds ratio; CI- Confidence interval**.**

Table 3 describes the multivariate analysis to identify predictors of length of stay in patients with PAH. Increased age, female gender, admission in a hospital in Midwest and alcohol use disorder were associated with decreased length of stay whereas presence of coagulopathy, and fluid and electrolyte disorders were associated with increased LOS.

**Table 3:** Multivariate regression analysis to determine factors associated with inpatient length of stay in pulmonary arterial hypertension patients.

Variable	β coefficient for length of hospitalization (95% Cl)	*p*
**Age, y**	−0.16 (−0.23 to −0.08)	**<0.0001**
18–44	−8.74 (−13.36 to −4.13)	**<0.0001**
45–64	−8.81 (−13.37 to −4.25)	**<0.0001**
65–84	−10.52 (−15.43 to −5.61)	**<0.0001**
≥85	−8.07 (−12.86 to −3.29)	**0.001**
**Female gender**	−3.47 (−6.71 to −0.24)	**0.035**
**Owner**		
Government	Reference	NA
Private, Not for profit	0.91 (−0.58–2.39)	0.232
Private, For profit	−1.60 (−4.03–0.84)	0.198
**Bed size**		
Small	Reference	NA
Medium	−6.54 (−14.30–1.21)	0.098
Large	−3.14 (−10.36–4.07)	0.392
**Race**		
White	Reference	NA
Black	3.94 (−1.43–9.32)	0.15
Hispanic	−0.22 (−1.71–1.26)	0.767
Asian	−1.05 (−3.81–1.71)	0.454
Native American	0.67 (−2.36–3.70)	0.663
**Region**		
Northeast	Reference	NA
Midwest	−3.42 (−6.50 to −0.33)	**0.03**
South	−1.63 (−4.71–1.45)	0.3
West	−0.71 (−3.77–2.34)	0.647
**Location**		
Rural location	Reference	NA
Urban, Non-teaching hospital	1.57 (−1.44–4.59)	0.305
Urban, Teaching hospital	0.82 (−1.90–3.55)	0.554
**Quartile classification of median household income for patient's ZIP Code**		
Quartile 1	Reference	NA
Quartile 2	1.62 (−0.88–4.13)	0.203
Quartile 3	0.75 (−1.30–2.80)	0.473
Quartile 4	0.68 (−1.61–2.96)	0.562
**Admission type**		
Non-elective	Reference	NA
Elective	−1.20 (−4.05–1.65)	0.41
**Admission Day**		
Weekday	Reference	NA
Weekend	0.25 (−2.48–2.98)	0.857
**Elixhauser comorbidities**		
Congestive heart failure	2.03 (0.001–4.06)	0.05
Cardiac arrhythmia	0.96 (−2.90–4.82)	0.625
Valvular heart disease	1.39 (−0.78–3.57)	0.209
Peripheral vascular disorder	2.82 (−0.59–6.23)	0.105
Other neurological disorders	1.44 (−7.47–10.35)	0.751
Uncomplicated diabetes	0.28 (−1.05–1.61)	0.681
Hypothyroidism	0.82 (−1.35–3.00)	0.457
Renal failure	1.62 (−2.93–6.16)	0.485
AIDS/HIV	−7.51 (−16.72–1.70)	0.11
Lymphoma	2.53 (−6.97–12.03)	0.601
Coagulopathy	5.83 (1.35–10.32)	**0.011**
Obesity	0.88 (−0.32–2.09)	0.151
Weight loss	7.28 (−3.15–17.71)	0.171
Fluid and electrolyte disorder	4.53 (1.07–7.99)	**0.01**
Deficiency anemia	0.57 (−3.69–2.55)	0.719
Alcohol abuse	−3.58 (−6.94 to −0.22)	**0.037**
Drug abuse	0.35 (−1.61–2.31)	0.724
Depression	0.29 (−1.33–1.92)	0.724

*P* < 0.05 was considered statistically significant. Bolded text represents the subheadings within the table and bolded numbers represent *p* < 0.05. CI- Confidence interval, AIDS/HIV- Acquired immunodeficiency syndrome/Human immunodeficiency virus.

**Fig. 3:**
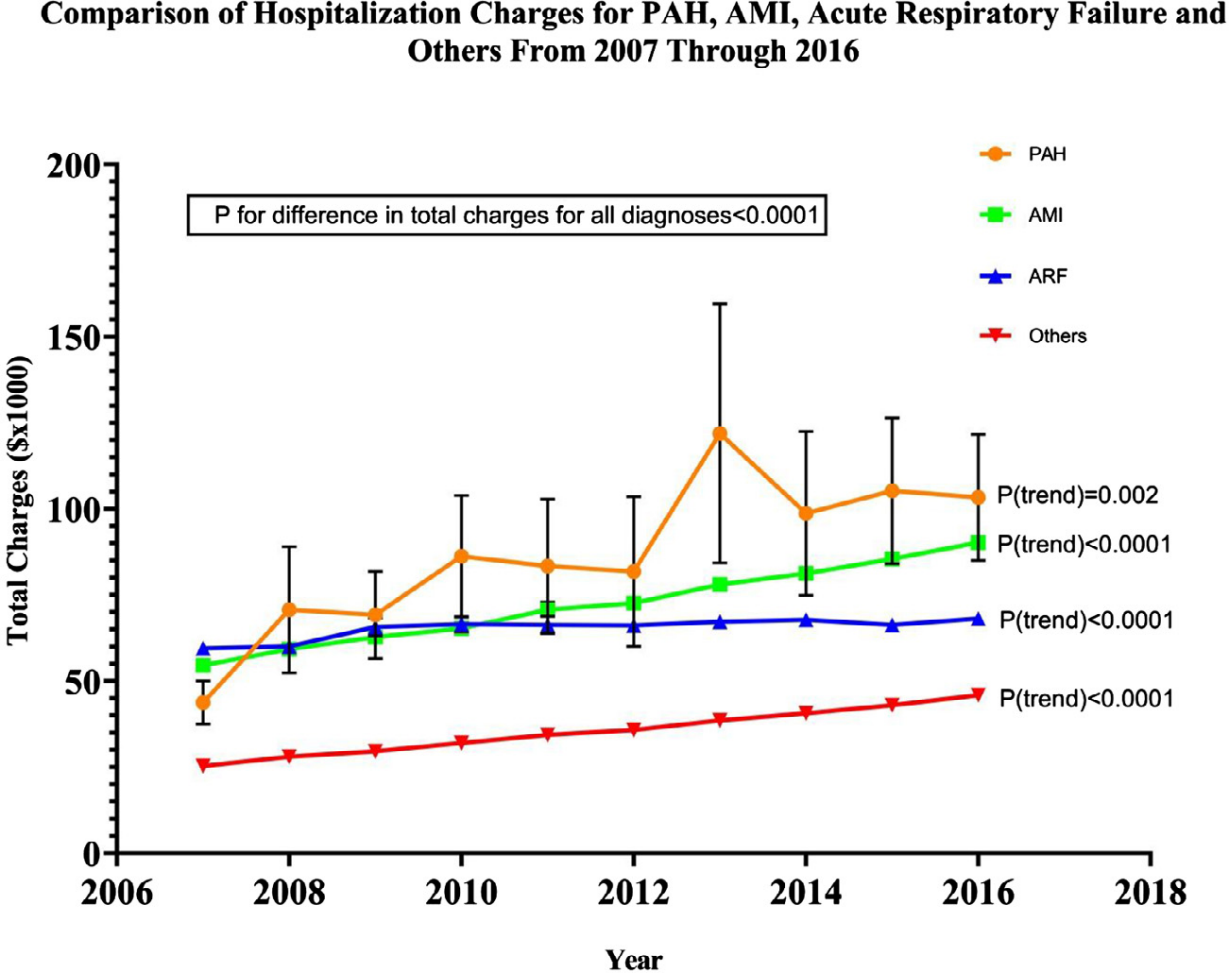
Temporal trends in the hospitalization charges of PAH compared to AMI, ARF and all other diagnoses.

Table 4 describes the comparison in the trends of the charges of the PAH-related hospitalizations compared to other common conditions like acute myocardial infarction, acute respiratory failure and others. While there had been a significant increase in the charges of all the conditions listed; the increase in the charges associated with pulmonary arterial hypertension related hospitalizations were significantly higher than the charges associated with acute myocardial infarction, acute respiratory failure and others (Fig. 3).

**Table 4:** Comparative trends in hospitalization charges for PAH, AMI, ARF and other principal diagnoses from 2007 through 2016.

Diagnosis	2007	2008	2009	2010	2011	2012	2013	2014	2015	2016	Ptrend
**PAH**	43.8 ± 6.3	70.7 ± 18.3	69.2 ± 12.6	86.2 ± 17.7	83.4 ± 19.5	81.8 ± 21.7	121.9 ± 37.6	98.7 ± 23.8	105.2 ± 21.2	103.3 ± 18.3	**0.002**
**AMI**	54.6 ± 1.7	59.3 ± 1.9	62.8 ± 1.9	65.3 ± 2	70.7 ± 2.2	72.6 ± 1.1	78 ± 1.1	81.2 ± 1.2	85.4 ± 1.2	90.2 ± 1.3	**<0.0001**
**ARF**	59.5 ± 2.1	60.1 ± 2.1	65.7 ± 2.6	66.5 ± 2.2	66.3 ± 2.5	66.1 ± 1.2	67.2 ± 1.2	67.8 ± 1.2	66.3 ± 1.1	68.1 ± 1.2	**<0.0001**
**Other diagnoses**	25.3 ± 0.6	28 ± 0.9	29.6 ± 0.8	32 ± 0.8	34.4 ± 1.1	35.8 ± 0.5	38.6 ± 0.5	40.6 ± 0.6	43 ± 0.6	45.9 ± 0.6	**<0.0001**

Continuous variables are expressed as mean ± SE. *p* < 0.05 was considered statistically significant. PAH- Pulmonary arterial hypertension; AMI- Acute myocardial infarction; ARF- Acute respiratory failure; SE- Standard error.

**Fig. 4:**
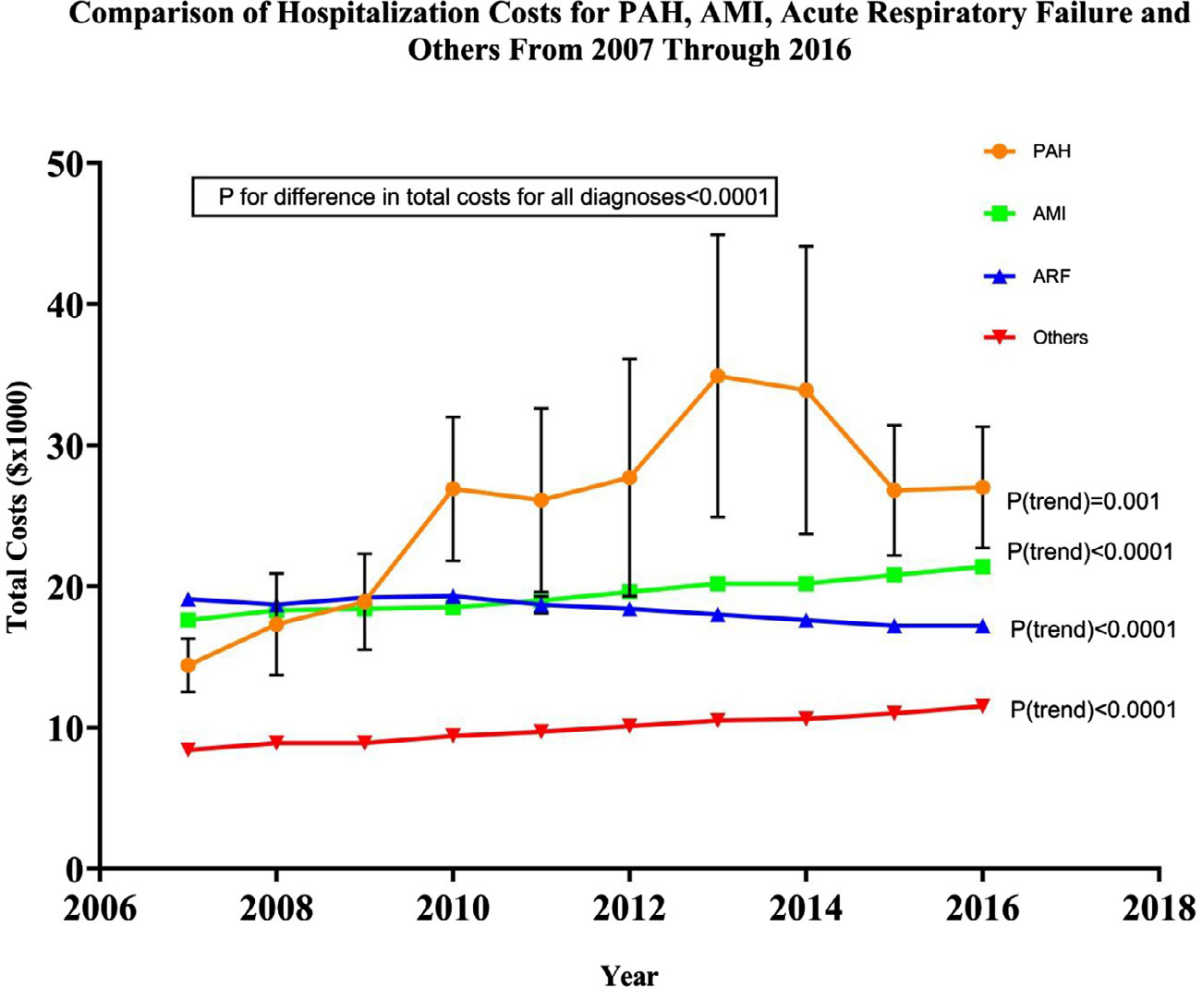
Temporal trends in the hospitalization costs of PAH compared to AMI, ARF and all other diagnoses.

Table 5 describes the comparison in the trends of the costs of the PAH-related hospitalizations compared to other common conditions like acute myocardial infarction, acute respiratory failure and others. While there had been a significant increase in the costs of all the conditions listed; the increase in the costs associated with pulmonary arterial hypertension related hospitalizations were significantly higher than the costs associated with acute myocardial infarction, acute respiratory failure and others (Fig. 4).

**Table 5:** Comparative trends in hospitalization costs for PAH, AMI, ARF and other principal diagnosis from 2007 through 2016.

Diagnosis	2007	2008	2009	2010	2011	2012	2013	2014	2015	2016	Ptrend
**PAH**	14.4 ± 1.9	17.3 ± 3.6	18.9 ± 3.4	26.9 ± 5.1	26.1 ± 6.5	27.7 ± 8.4	34.9 ± 10	33.9 ± 10.2	26.8 ± 4.6	27.0 ± 4.3	**0.001**
**AMI**	17.6 ± 0.3	18.3 ± 0.3	18.4 ± 0.3	18.5 ± 0.4	19 ± 0.4	19.6 ± 0.2	20.2 ± 0.2	20.2 ± 0.2	20.8 ± 0.2	21.4 ± 0.2	**<0.0001**
**ARF**	19.1 ± 0.5	18.7 ± 0.5	19.2 ± 0.5	19.3 ± 0.5	18.7 ± 0.6	18.4 ± 0.3	18 ± 0.3	17.6 ± 0.2	17.2 ± 0.2	17.2 ± 0.2	**<0.0001**
**Other diagnosis**	8.4 ± 0.2	8.9 ± 0.2	8.9 ± 0.2	9.4 ± 0.2	9.7 ± 0.2	10.1 ± 0.1	10.5 ± 0.1	10.6 ± 0.1	11 ± 0.1	11.5 ± 0.1	**<0.0001**

Continuous variables are expressed as mean ± SE. *p* < 0.05 was considered statistically significant. PAH- Pulmonary arterial hypertension; AMI- Acute myocardial infarction; ARF- Acute respiratory failure; SE- Standard error.

Fig. 1 is a graphical representation of the distribution of Elixhauser comorbidities (as was also described in Table 1) in patients with PAH.

Fig. 2 is a graphical representation of the distribution of other specific comorbidities (as was also described in Table 1) in patients with PAH.

Fig. 3 is a graphical representation of the temporal trends in the hospitalization charges of PAH compared to acute myocardial infarction, acute respiratory failure and all other diagnoses from 2007 through 2016 (as described in Table 4).

Bars indicate standard errors and solid lines indicate mean charges for respective diagnosis. *P* < 0.05 was considered statistically significant. PAH- Pulmonary arterial hypertension; AMI- Acute myocardial infarction; ARF- Acute respiratory failure.

Fig. 4 is a graphical representation of the temporal trends in the hospitalization costs of PAH compared to acute myocardial infarction, acute respiratory failure and all other diagnoses from 2007 through 2016 (as described in Table 5).

Bars indicate standard errors and solid lines indicate mean charges for respective diagnosis. *P* < 0.05 was considered statistically significant. PAH- Pulmonary arterial hypertension; AMI- Acute myocardial infarction; ARF- Acute respiratory failure.

## 2 Experimental design, materials and methods

### 2.1 Data source

The Healthcare Utilization Project (HCUP) is a family of databases developed through a Federal-State-Industry partnership and is sponsored by the Agency for Healthcare Research and Quality. National Inpatient Sample (NIS) database is an HCUP database that is the largest, publicly available, all-payer administrative claims database of inpatient hospitalizations in the US. It represents a random, 20% stratified sample of all inpatient hospitalizations from approximately 1000 non-federal hospitals in 46 states (representing >97% of the total US population) and includes approximately 7 to 8 million hospitalizations per year [2]. A discharge weight is provided for each patient discharge record to represent the relative proportion of the total US inpatient hospital population for each record, allowing for calculation of national estimates [3]. Therefore, the PAH cohort represented in this study is broadly representative of PAH population within US. The NIS database includes de-identified information on patient demographics, and clinical data including primary and secondary discharge diagnoses, comorbidities, and outcomes for each sampled hospitalization. All diagnoses and comorbidities are available in the NIS database as International Classification of Diseases, Ninth Revision, Clinical Modification (ICD-9-CM) codes until September 30, 2015, and as International Classification of Diseases, Tenth Revision, Clinical Modification/Procedure Coding System (ICD-10-CM/PCS) from October 2015 onwards. Since this is an analysis of publicly available de-identified data, our Institutional Review Board guidelines stipulate that board approval of the study and need for informed consent are waived.

### 2.2 Data availability

HCUP NIS data are available from the AHRQ, Rockville, MD (https://www.hcup-us.ahrq.gov/nisoverview.jsp). HCUP data are available to all researchers following a standard application process and signing of a data use agreement. The authors confirm that they had no special access to the data used in this study (2007–2016 HCUP NIS). The authors paid a fee to access the NIS data used in this study, in accordance with the fee schedule posted in the HCUP Central Distributor, the entity that accepts, processes, and fulfills applications for the purchase of HCUP databases and manages data use agreements (DUAs) for all data users (https://www.hcup-us.ahrq.gov/tech_assist/centdist.jsp). Researchers interested in purchasing and using HCUP databases will be required to complete the Web-based HCUP DUA (https://www.hcup-us.ahrq.gov/tech_assist/dua.jsp) and read and sign the HCUP DUA. Further instructions for submitting an application for purchasing HCUP Databases can be found at (https://www.distributor.hcup-us.ahrq.gov).

### 2.3 Validation and quality control

Annual data quality assessments of NIS are performed to maintain the internal validity of the database. Estimates from the NIS are compared with the American Hospital Association Annual Survey Database, National Hospital Discharge Survey from the National Center for Health Statistics, and the MedPAR inpatient database from Centers for Medicare and Medicaid Services [4].

### 2.4 Study population

We identified all patients in the NIS database from January 1, 2007, through December 31, 2016, with a primary discharge diagnosis of PAH using an ICD-9-CM primary diagnosis code of 416.0 and ICD-10-CM/PCS primary diagnosis code of I27.0. Discharge records suggestive of secondary causes of pulmonary hypertension (WHO Category II-V) were excluded. These included left heart (systolic or diastolic) failure, chronic obstructive pulmonary disease, interstitial lung disease, mitral or aortic valve disease, atrial flutter/fibrillation, coronary artery disease, complicated hypertension and diabetes, end stage renal disease, metastatic cancer and paralysis. This yielded a final cohort of 6162 records with PAH as primary diagnosis as described in the related original article [1].

### 2.5 Covariates

For each discharge record with primary diagnosis of PAH, we obtained the following variables: patient demographics, comorbidities, admission characteristics (elective vs. non-elective, primary payer, disposition status), and hospital characteristics (US region, government vs. private, teaching vs. non-teaching, hospital bed-size). Patient's income status was defined per HCUP's quartile classification of the estimated median household income of residents in the patient's ZIP Code, indicating the poorest to wealthiest populations [5]. Primary payer status was categorized as Medicare, Medicaid, private insurance and uninsured. Disposition at discharge was categorized as routine, short term hospital, nursing home or rehabilitation and home health care[5]. Hospital size was defined as small, medium, and large as per HCUP criteria [6].

Comorbidity burden was assessed using Elixhauser comorbidities and index based on coding algorithms developed by Elixhauser et al. [7] and Quan et al. [8] and have been previously validated as predictors of outcomes in administrative databases [9, 10]. We also compared distribution of few other specific inpatient comorbidities that are clinically pertaining to PAH patients (e.g. congenital heart disease, syncope, cardiogenic shock, cirrhosis, portal hypertension, hepatitis C, respiratory failure, pneumonia, kidney disease, acute cerebrovascular disease). These comorbidities were identified using valid ICD-9-CM and ICD-10-CM/PCS codes [11, 12].

The raw hospital charges provided in the NIS database represent the amount of money billed by the hospitals for services rendered but it does not provide the amount of money hospital services actually cost or the specific amounts that hospitals received in payment. The HCUP Cost-to-Charge Ratios enable this conversion to provide actual expenses incurred in the production of hospital services, such as wages, supplies, and utility costs. The cost of each inpatient stay was estimated by multiplying the total hospital charge with the cost-to-charge ratio. We report the charges and cost for each year after adjustment for inflation with reference to 2016 U.S. dollar value, using the latest Consumer Price Index data (http://www.bls.gov/data/inflation_calculator.htm).

### 2.6 Outcomes

Our primary outcomes included total hospitalizations with the primary discharge diagnosis of PAH, associated length of stay, all-cause inpatient mortality and charges (and costs) per hospitalization. As secondary outcomes, we examined the predictors of inpatient mortality and prolonged length of stay along with trends in the charges and costs of PAH hospitalization compared to other common causes of hospitalization like acute myocardial infarction, acute respiratory failure and all other diagnoses.

### 2.7 Statistical analysis

National estimates for total number of discharges with primary diagnosis of PAH per year (from 2007 through 2016) were generated from the NIS database using trend weights and published HCUP methods [13]. Continuous variables are reported as mean ± standard error, and categorical variables are reported as frequency (percentages). Trends were analyzed using linear regression for continuous variables. Multivariate regression models were generated to identify factors associated with all-cause inpatient mortality and length of stay. The clinically relevant variables and those with *p* < 0.3 in the univariate analysis, were included in the corresponding multivariable-adjusted models. All analyses in the study were performed according to recommended AHRQ/HCUP methods to account for complex survey design, cluster, stratification and weighting, and in agreement with the best research practices for conducting research using the NIS database. All statistical analysis was completed using STATA 15.0 (StataCorp, College Station, TX, USA). *P*value < 0.05 was considered for statistical significance.

## CRediT Author Statement

Conceptualization, Methodology, Pre-processing: AC, MK, RLB

Writing-Reviewing and Editing: AC, MK, PC, TT, AR, RLB

## Ethics Statement

This is an analysis of publicly available de-identified patient data. Therefore our Institutional Review Board guidelines stipulate that board approval of the study and need for informed consent are waived.

## Declaration of Competing Interest

All authors declare that they have no known competing financial interests or personal relationships which have, or could be perceived to have, influenced the work reported in this article.
